# Total Phenolic Contents and Antioxidant Capacities of Selected Chinese Medicinal Plants

**DOI:** 10.3390/ijms11062362

**Published:** 2010-06-01

**Authors:** Feng-Lin Song, Ren-You Gan, Yuan Zhang, Qin Xiao, Lei Kuang, Hua-Bin Li

**Affiliations:** Department of Nutrition, School of Public Health, Sun Yat-Sen University, Guangzhou 510080, China; E-Mails: sflin1986@163.com (F.-L.S.); ganry_zsu@yahoo.cn (R.-Y.G.); fly198013@yahoo.com.cn (Y.Z.); hbycxiaoq@163.com (Q.X.); kwung_1121@hotmail.com (L.K.)

**Keywords:** medicinal plant, phenolic content, antioxidant capacity

## Abstract

Antioxidant capacities of 56 selected Chinese medicinal plants were evaluated using the Trolox equivalent antioxidant capacity (TEAC) and ferric reducing antioxidant power (FRAP) assays, and their total phenolic content was measured by the Folin-Ciocalteu method. The strong correlation between TEAC value and FRAP value suggested that the antioxidants in these plants possess free radical scavenging activity and oxidant reducing power, and the high positive correlation between antioxidant capacities and total phenolic content implied that phenolic compounds are a major contributor to the antioxidant activity of these plants. The results showed that *Dioscorea bulbifera, Eriobotrya japonica, Tussilago farfara* and *Ephedra sinica* could be potential rich sources of natural antioxidants.

## 1. Introduction

Free radicals are produced as a part of normal metabolic processes. They are extremely reactive, highly unstable and potentially damaging transient chemical species. Under physiological conditions, the cellular redox state is tightly controlled by antioxidant enzymatic systems and chemical scavengers such as endogenous enzymes, dietary antioxidants as well as some hormones [1]. However, an overproduction of free radicals on one side and (or) a deficiency of enzymatic and non-enzymatic antioxidants on the other, will lead to significant increased production of these radicals, which overwhelm the antioxidant defense and impose oxidative stress on the physiological system [2]. The excess oxidative stress can cause damage to cellular lipids, proteins, or DNA, inhibiting their normal functions. Oxidative stress has been implicated in many degenerative diseases such as atherosclerosis, coronary heart diseases, aging and cancer [3–5]. Therefore, minimizing oxidative stress will promote our physical condition and prevent some degenerative diseases in which free radicals are involved. In addition, antioxidants have been widely used in the food industry to prolong the shelf life. Nowadays, natural antioxidants, due to their limited sources and high price, are not widely used. Synthetic antioxidants, such as butylated hydroxytoluene and butylated hydroxyanisole, are commonly used in the food industry. However, there is a widespread agreement that synthetic antioxidants need to be replaced with natural antioxidants because some synthetic antioxidants have shown potential health risks and toxicity, most notably possible carcinogenic effects [6,7]. Therefore, it is of great importance to find new sources of safe and inexpensive antioxidants of natural origin in order to use them in foods and pharmaceutical preparations to replace synthetic antioxidants.

The best health and nutrition results can be achieved not only from the consumption of fruits and vegetables with high antioxidant capacities, but also from medicinal herbs and plants [8]. Several studies indicated that some Chinese medicinal plants possess more potent antioxidant activity than common fruits and vegetables, and phenolic compounds were a major contributor to the antioxidant activity of these plants [9,10]. In particular, a group of Chinese medicinal plants is traditionally used for prevention and treatment of cold, flu and cough. Furthermore, some of these plants also possessed significant anticancer, anti-inflammation, anti-allergic and antimicrobial activities [11]. On the other hand, these beneficial effects of medicinal plants could be partly attributed to their antioxidant and free radical scavenging activities [9,11,12]. This prompted us to hypothesize that these plants could contain rich antioxidant compounds. In this study, 56 medicinal plants were selected to assess their antioxidant capacities and total phenolic contents.

The aims of this study were to find new sources of safe and inexpensive antioxidants from 56 selected Chinese medicinal plants using the ferric reducing antioxidant power (FRAP) and Trolox equivalent antioxidant capacity (TEAC) assays, to determine their total phenolic contents using the Folin-Ciocalteu method, and to investigate the relationship between antioxidant capacity and total phenolic content.

## 2. Results and Discussion

### 2.1. ABTS^•+^ Radical Scavenging Activity

Trolox equivalent antioxidant capacity (TEAC) assay is one of the most commonly employed methods for determining antioxidant capacity. The TEAC assay measures the ability of a compound to scavenge ABTS^•+^ radicals, and is widely used to screen antioxidant activity of fruits, vegetables, foods and plants, and is applicable to both lipophilic and hydrophilic antioxidants [13]. In particular, it is recommended to be used for plant extracts because the long wavelength absorption maximum at 734 nm eliminates color interference in plant extracts. In this study, antioxidant capacities of the methanol extracts from 56 selected Chinese medicinal plants were measured using the TEAC assay and the results are shown in Table 1. The plants, in general, showed high antioxidant capacities, ranging from 0.61 to 708.73 μmol Trolox/g. The difference of antioxidant capacities was very large, up to 1162-fold. *Dioscorea bulbifera* showed the highest antioxidant capacity (708.73 μmol Trolox/g), followed by *Eriobotrya japonica* (326.87 μmol Trolox/g), *Tussilago farfara* (217.62 μmol Trolox/g), *Ephedra sinica* (197.69 μmol Trolox/g) and *Ardisia japonica* (164.17 μmol Trolox/g), whereas *Pinellia ternata* exhibited the lowest antioxidant capacity (0.61 μmol Trolox/g).

### 2.2. Ferric Reducing Antioxidant Power

The antioxidant capacity of the plant extract largely depends on both the composition of the extract and the test system. It can be influenced by a large number of factors, and can not be fully evaluated by one single method. It is necessary to perform more than one type of antioxidant capacity measurement to take into account the various mechanisms of antioxidant action [14]. Therefore, antioxidant capacities of 56 selected Chinese medicinal plants were also evaluated using the Ferric reducing antioxidant power (FRAP) assay. In this assay, the antioxidant capacity is measured on the basis of the ability to reduce ferric(III) ions to ferrous(II) ions. The FRAP assay is a simple method, and can be applied to both aqueous and alcohol extracts of plants. As seen from Table 1, the antioxidant capacities of these plants ranged from 0.15 μmol Fe^2+^/g to 856.92 μmol Fe^2+^/g, with large differencse in antioxidant capacities of up to 5713-fold. *Dioscorea bulbifera* possessed the highest antioxidant capacity (856.92 μmol Fe^2+^/g), followed by *Tussilago farfara* (455.64 μmol Fe^2+^/g), *Eriobotrya japonica* (437.40 μmol Fe^2+^/g), *Ephedra sinica* (388.68 μmol Fe^2+^/g) and *Arctium lappa* (223.68 μmol Fe^2+^/g). *Sargassum fusiforme* showed the lowest antioxidant capacity (0.15 μmol Fe^2+^/g) among these plants.

As shown in Table 1, *Dioscorea bulbifera, Eriobotrya japonica, Tussilago farfara* and *Ephedra sinica* had the highest antioxidant capacities among the 56 plants based on a combinative consideration of the results obtained by FRAP and TEAC assays. They are potential sources of natural antioxidants for preparation of crude extracts or further isolation and purification of antioxidant components. Especially, if the crude extract is nontoxic after the toxicological assessment, further isolation and purification of antioxidant components is not necessary because health benefits of the extract might be from additive and synergistic effects of phytochemicals in the extract [15]. At this condition, the extract can be directly used for consumption at home, and also may be used as food additive in food industry.

*Dioscorea bulbifera* is used to prevent and treat several diseases, such as sore throat, Struma, tumors, diabetes and leprosy [11,16]. To our knowledge, there was no prior report as to the antioxidant activity of this plant. The present study provided valuable preliminary data by demonstrating its high antioxidant capacity, and isolation and characterization of its individual active components await further comprehensive studies. Caryatin, (+)-catechin, myricetin, kaempferol-3,5-dimethylether, quercetin-3-*O*-galactopyranoside, diosbulbin B, myricetin-3-*O*-galactopyranoside and myricetin-3-*O*-glucopyranoside were identified from this plant [16,17], and some of them could be antioxidant components.

*Eriobotrya japonica* is traditionally used for the prevention and treatment of cough and asthma, which contained some phenolics and triterpenes that exhibited beneficial effects of anticancer, anti-inflammation, hypoglycemia and hypolipidemia [11,18]. In this study, antioxidant activity of *Eriobotrya japonica* was assessed for the first time. It showed very high antioxidant capacity, and is a potential source of natural antioxidant. Ursolic acid, oleanolic acid, maslinic acid, malic acid, amygdalin, saponins, hyperin and catechin were identified from this plant [11,18], and some of them could be antioxidant components.

*Tussilago farfara* is used for the treatment of cough, bronchitis and asthmatic disorders, and has antimicrobial activity [11]. The antioxidant potency of this plant demonstrated in the present study was agreement with the previous study [19]. Faradiol, tussilagin, angelic acid, hyperin, bauerend, arnidiol, senecionine, rutin and quercetin-glycosides were identified from this plant [11,19], and some of them could be antioxidant components.

In the present study, another new candidate for a natural antioxidant was *Ephedra sinica*, which is used for treatment of multiple symptoms of cold and allergy. It is also useful to control body weight, treat fulminant hepatic failure, and to alleviate inflammatory responses [11,20,21]. Ephedrine and pseudoephedrine are its two primary active ingredients, which possess a variety of biological activities. However, its antioxidant components are still unclear, and further research is needed to isolate and characterize compounds responsible for the antioxidant effects.

**Figure 1.**
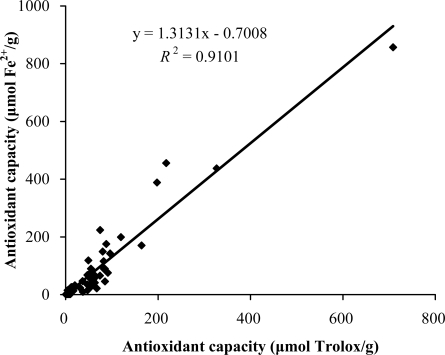
Correlation between the antioxidant capacities measured by the FRAP and TEAC assays.

As shown in Figure 1, the antioxidant capacities obtained from the TEAC assay were in good accordance with those obtained from the FRAP assay (*R*^2^ = 0.9348), which implies that the antioxidants in these plants were able to scavenge free radicals (ABTS^•+^) and reduce oxidants (ferric ions). However, some plants, such as *Fverticillata Willd* and *Arctium lappa*, possessed relatively high radical scavenging activities, but low oxidant reducing activities. These differences might result from the different antioxidant mechanisms of the antioxidants they contain, which is worth to be studied further.

### 2.3. Total Phenolic Content

Total phenolic content of the 56 selected plants were measured using the Folin-Ciocalteu method, and the results are shown in Table 1. As seen from Table 1, the total phenolic content of these plants ranged from 0.12 to 59.43 mg GAE/g, with large differences between the plants of up to 495-fold. *Dioscorea bulbifera* showed the highest phenolic content (59.43 mg GAE/g), followed by *Eriobotrya japonica* (31.47 mg GAE/g), *Tussilago farfara* (30.03 mg GAE/g), *Ephedra sinica* (27.70 mg GAE/g), and *Pueraria lobata* (24.01 mg GAE/g), whereas *Pinellia ternata* showed the lowest phenolic content (0.12 mg GAE/g) of these plants. Phenolic compounds are plant metabolites characterized by the presence of several phenol groups. Some of them are very reactive in neutralizing free radicals by donating a hydrogen atom or an electron, chelating metal ions in aqueous solutions [22]. Besides, the phenolic compounds possess multiple biological properties such as antitumor, antimutagenic and antibacterial properties, and these activities might be related to their antioxidant activity [23].

### 2.4. Correlation between Antioxidant Capacity and Total Phenolic Content

**Figure 2.**
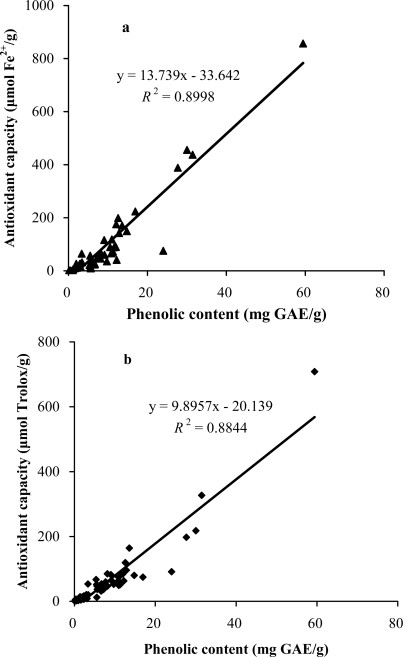
Correlation between the antioxidant capacity and total phenolic content of the selected plants. Antioxidant capacity was measured by the FRAP assay (a) and TEAC assay (b). GAE: gallic acid equivalents.

Despite the presence of a wide range of the total antioxidant capacities and total phenolic contents among the selected plants, linear positive relationships could be found between the TEAC value and total phenolic content, as well as between the FRAP value and the total phenolic content, as shown in Figure 2.

The strong correlations between the results using the two methods of measuring antioxidant capacity and the total phenolic content showed that phenol compounds largely contribute to the antioxidant activities of these plants, and therefore could play an important role in the beneficial effects of these plants. The results were in accordance with other researches [9,24]. However, there were some plants, such as *Perilla frutescens*, which exhibited relatively high antioxidant capacity, but did not contain comparable phenolic content. This suggests the presence of other antioxidant compounds in some of the medicinal plants, which meets the general agreement that the extracts of Chinese medicinal plants often contain complex mixtures of different kinds of active compounds, and the contribution from compounds other than phenolics should not be neglected.

## 3. Experimental Section

### 3.1. Chemicals and Plant Materials

Trolox, 2,2′-azinobis(3-ethylbenothiazoline-6-sulfonic acid) diammonium salt (ABTS), Folin–Ciocalteu’s phenol reagent, 2,4,6-tri(2-pyridyl)-*s*-triazine (TPTZ) and gallic acid were purchased from Sigma-Aldrich (St. Louis, MO). Potassium persulfate, iron (III) chloride 6-hydrate, iron (II) sulfate 7-hydrate, acetic acid, sodium carbonate, acetic acid, sodium acetate, hydrochloric acid and methanol were obtained from Tianjing Chemical Factory (Tianjing, China). All the chemicals and reagents used in the experiments were of analytical grade.

Fifty-six selected Chinese medicinal plants used in this investigation were obtained from a famous vendor of traditional Chinese medicines, Beijing Tong-Ren-Tang drug retail outlet in Guangzhou of China.

### 3.2. Sample Preparation

The dry plant sample was ground to fine powder in a mill, and 0.5 g of powder of each sample was treated with 10 mL of methanol-water (8:2, v/v) in a shaking water bath at 35 °C for 24 h as described [9]. The mixture was then cooled to room temperature and centrifuged at 4,000 rpm for 10 min. The supernatant was recovered for the determination of the antioxidant capacity and total phenolic content. All the experiments were carried out in triplicate.

### 3.3. Trolox Equivalent Antioxidant Capacity Assay

The TEAC assay was carried out according to the method of Re *et al*. [25]. Firstly, to produce the radical cation ABTS^•+^, 7 mmol/L ABTS salt and 2.45 mmol/L potassium persulfate were mixed in a volume ratio of 1:1, the reaction mixture was allowed to stand in the dark for 16 h at room temperature and was used within two days of preparation. The ABTS^•+^ radical solution was diluted with ethanol to an absorbance of 0.7 ± 0.05 at 734 nm. All samples were diluted approximately to provide 20–80% inhibition of the blank absorbance. One hundred microliters of the diluted sample was mixed with 3.8 mL ABTS^•+^ working solution, and the reaction mixture was left at room temperature to react for 6 min, and then the absorbance at 734 nm was taken using the ultraviolet spectrophotometer [26]. Trolox solution was used as a reference standard, and the results were expressed as μmol Trolox/g dry weight of herbal material.

### 3.4. Ferric Reducing Antioxidant Power Assay

Measurement of ferric reducing antioxidant power of the herbal extract was carried out based on the procedure of Benzie and Strain [27]. Firstly, sodium acetate buffer (300 mmol/L, pH 3.6), 10 mmol/L TPTZ solution (40 mmol/L HCl as solvent) and 20 mmol/L iron (III) chloride solution were mixed in a volume ratio of 10:1:1 to generate FRAP reaction solution, which should be prepared fresh daily and be warmed to 37 °C in a water bath before use. Then 100 μL of the diluted sample was added to 3 mL of the FRAP reaction solution. After 4 min of reaction, the absorbance of the reaction mixture was recorded at 593 nm [24]. The standard curve was constructed using FeSO_4_ solution, and the results were expressed as μmol Fe(II)/g dry weight of herbal material.

### 3.5. Determination of Total Phenolic Content

Total phenolic content was determined with the Folin-Ciocalteu reagent according to a procedure described by Singleton and Rossi [28]. Briefly, 0.50 mL of the diluted sample was reacted with 2.5 mL of 0.2 mol/L Folin-Ciocalteu reagent for 4 min, and then 2 mL saturated sodium carbonate solution (about 75 g/L) was added into the reaction mixture. The absorbance readings were taken at 760 nm after incubation at room temperature for 2 h. Gallic acid was used as a reference standard, and the results were expressed as milligram gallic acid equivalent (mg GAE)/g dry weight of herbal material.

### 3.6. Statistical Analysis

All the experiments were carried out in triplicate, and the results were expressed as mean ± SD (standard deviation). Statistical analysis was performed using SPSS 13.0 and Excel 2003. The *p* value less than 0.05 was considered to be statistically significant.

## 4. Conclusions

The antioxidant capacities and total phenolic contents of 56 selected medicinal plants were evaluated, and the potential antioxidant activities of many plants were assessed for the first time. Positive correlations between antioxidant capacity and total phenolic content suggest that the antioxidant activities of the medicinal plants can be mainly ascribed to their phenol compounds. A strong correlation between TEAC value and FRAP value implies that antioxidants in these plants possess free radical scavenging activity and oxidant reducing power. The results indicate that *Dioscorea bulbifera, Eriobotrya japonica, Tussilago farfara* and *Ephedra sinica* are the most potent, since they exhibited the highest scavenging activity against ABTS^•+^ radical, outstanding reducing power and plenteous phenolic contents among 56 studied plants. Besides their strong antioxidant activities, their low toxicities, wide distributions and medicinal functions [11] all make them promising sources of natural antioxidants and other bioactive compounds in food and pharmaceutical industries.

## Figures and Tables

**Table 1.** Antioxidant capacities and total phenolic contents of 56 Chinese medicinal plants.

**Scientific name**	**TEAC value(μmol Trolox/g)**	**FRAP value(μmol Fe^2+^/g)**	**Phenolic content (mg GAE/g)**
*Angelica dahurica* Benth. et Hook	20.79 ± 3.67	27.36 ± 0.49	2.94 ± 0.11
*Arctium lappa* L.	74.66 ± 0.53	223.68 ± 8.28	16.94 ± 1.7
*Ardisia japonica* (Horrst) Bl.	164.17 ± 2.39	170.2 ± 4.39	13.58 ± 0.03
*Arisaema consanguineum* Schott	0.78 ± 0.14	1.05 ± 0.18	0.24 ± 0.02
*Aster tataricus* L. F.	47.38 ± 1.43	14.77 ± 0.89	5.56 ± 0.21
*Bambusa breviflora* Munro	82.46 ± 1.03	115.74 ± 3.91	9.03 ± 0.26
*Brassica alba* L. Boiss	53.51 ± 3.3	64.87 ± 2.55	3.34 ± 0.37
*Bupleurum chinense* D. C.	19.93 ± 0.29	32.05 ± 2.22	3.41 ± 0.21
*Centipeda minima* (L.) A. Br. Et Ascher	16.87 ± 2.4	13.31 ± 0.61	2.34 ± 0.03
*Changiumsmyrnioides* Wolff	2.07 ± 0.07	0.35 ± 0.02	0.50 ± 0.01
*Chrysanthemum* indicum L.	51.91 ± 0.84	72.16 ± 4.88	11.28 ± 0.10
*Chrysanthemum morifolium* Ramat.	80.04 ± 2.55	149.24 ± 2.9	14.79 ± 1.41
*Cimicifuga foetida* L.	119.50 ± 1.43	199.08 ± 0.5	12.57 ± 0.17
*Cinnamomum cassia* Presl	52.75 ± 0.55	35.59 ± 1.16	9.71 ± 0.10
*Cynanchum stauntoni* (Decne.) Schltr.	14.24 ± 0.38	9.77 ± 1.06	1.40 ± 0.09
*Dioscorea bulbifera* L.	708.73 ± 3.7	856.92 ± 3.99	59.43 ± 1.03
*Elsholtziasplendens* Wakaiex	59.84 ± 3.09	52.55 ± 4.99	7.71 ± 0.03
*Ephedra sinica* Seapf	197.69 ± 3.36	388.68 ± 9.58	27.70 ± 0.89
*Equisetum hiema* L.	10.66 ± 1.04	13.79 ± 0.72	2.68 ± 0.05
*Eriobotrya japonica* (Thunb.) Lindl.	326.87 ± 7.17	437.4 ± 7.42	31.47 ± 0.48
*Fritillaria cirrhosa* D. Don	2.57 ± 0.04	0.29 ± 0.05	0.96 ± 0.07
*Fverticillata* Willd.	9.83 ± 0.21	0.91 ± 0.13	1.07 ± 0.09
*Ginkgo biloba* L. (fruit)	11.63 ± 0.31	11.67 ± 1.01	2.14 ± 0.01
*Ginkgo biloba* L. (leaf)	82.89 ± 1.06	88.76 ± 5.01	11.55 ± 0.18
*Gleditsia sinensis* Lam.	54.14 ± 2.92	26 ± 2.38	6.68 ± 0.23
*Inula britannica* L.	96.12 ± 2.20	142.31 ± 5.13	12.83 ± 0.56
*Laminaria japonica* Aiesch	6.86 ± 0.64	0.33 ± 0.06	0.36 ± 0.03
*Lepidium apetalum* Willd	47.23 ± 0.73	34.64 ± 4.13	5.91 ± 0.08
*Ligusticum sinense* Oliv	84.71 ± 0.93	89.84 ± 3.70	11.99 ± 0.05
*Magnolialilifora* Desr	49.19 ± 4.13	118.53 ± 11.61	10.98 ± 0.31
*Mentha haplocalyx* Briq	87.80 ± 7.80	175.06 ± 3.94	12.08 ± 0.26
*Momordica grosvenor*i Swingle	63.17 ± 0.30	41.28 ± 3.55	12.22 ± 1.27
*Morus alba* L. (bark of root)	67.22 ± 5.07	21.67 ± 1.20	5.34 ± 0.09
*Morus alba* L. (leaf)	74.19 ± 1.67	65.79 ± 4.11	10.94 ± 0.21
*Notopterygiumincisum* Ting	62.94 ± 4.32	66.80 ± 2.03	10.86 ± 0.31
*Oraxylum indicum* (L.) Vent	85.20 ± 1.16	45.64 ± 2.17	8.15 ± 0.61
*Perilla frutescens* (L.) Britt. (leaf)	36.47 ± 1.81	46.8 ± 2.14	7.17 ± 0.05
*Perilla frutescens* (L.) Britt. (seed)	13.71 ± 1.19	26.29 ± 3.01	1.96 ± 0.10
*Perilla frutescens* (L.) Britt. (stem)	11.91 ± 0.67	25.34 ± 0.82	2.8 ± 0.07
*Peucedanum praeruptorum* Dunn	4.20 ± 0.15	14.78 ± 1.95	1.6 ± 0.15
*Physalis alkekengi* L.	64.29 ± 2.59	60.42 ± 4.49	9.12 ± 0.31
*Pinellia ternata* (Thunb.) Breit	0.61 ± 0.05	0.46 ± 0.02	0.12 ± 0.01
*Platycodon grandiflorus* Jacq.	6.42 ± 0.15	5.26 ± 0.73	1.15 ± 0.05
*Prunus armeniaca* L.var. ansu Maxim.	4.18 ± 0.05	0.41 ± 0.04	0.58 ± 0.03
*Pueraria lobata* (Willd.) Ohwi (root)	8.51 ± 0.37	13.87 ± 1.66	3.11 ± 0.09
*Pueraria lobata* (Willd.) Ohwi (flower)	91.52 ± 2.07	75.55 ± 4.37	24.01 ± 1.76
*Saposhnikovia divaricata* Turcz.	6.39 ± 0.81	14.22 ± 1.12	2.31 ± 0.23
*Sargassum fusiforme* Turn.	3.89 ± 0.52	0.15 ± 0.02	0.18 ± 0.01
*Schizonepeta ternnuifolia* (Benth) Briq	47.13 ± 1.39	67.97 ± 3.76	8.17 ± 0.03
*Spirodela polyrrhiza* (L.) Schleid.	54.84 ± 3.21	89.55 ± 4.89	10.53 ± 0.23
*Stemona sessilifolia* (Miq.) Franch	12.21 ± 0.61	22.87 ± 3.93	5.55 ± 0.11
*Sterculia scaphigera* Wall.	52.26 ± 0.87	57.28 ± 9.81	5.49 ± 0.12
*Trichosanthes Ririlowii* Maxim	11.01 ± 0.32	9.53 ± 0.97	1.66 ± 0.17
*Tussilago farfara* L.	217.62 ± 5.35	455.64 ± 5.03	30.03 ± 0.19
*Vitex yotundifolia* L.	37.18 ± 1.61	9.35 ± 1.09	5.66 ± 0.16
*Xanthium sibiricum* Patr. ex Widd	31.42 ± 0.83	23.63 ± 2.86	6.6 ± 0.22
